# Spatial distribution and determinants of intermittent preventive treatment for malaria during pregnancy: a secondary data analysis of the 2019 Ghana malaria indicators survey

**DOI:** 10.1186/s12884-024-06566-0

**Published:** 2024-05-20

**Authors:** Jean Claude Ndayishimiye, Philip Teg-Nefaah Tabong

**Affiliations:** Department of Social and Behavioural Sciences, University of Ghana School of Public Health, Legon, Accra Ghana

**Keywords:** Intermittent preventive treatment, Malaria during pregnancy, Spatial distribution, Determinants, Ghana

## Abstract

**Background:**

Malaria during pregnancy is associated with poor maternal, foetal, and neonatal outcomes. To prevent malaria infection during pregnancy, the World Health Organization recommended the use of intermittent preventive therapy with sulfadoxine-pyrimethamine (IPTp-SP) in addition to vector control strategies. Although Ghana’s target is to ensure that all pregnant women receive at least three (optimal) doses of SP, the uptake of SP has remained low; between 2020 and 2022, only 60% of pregnant women received optimal SP during their most recent pregnancy. This study sought to map the geospatial distribution and identify factors associated with SP uptake during pregnancy in Ghana.

**Methods:**

Secondary data analysis was conducted using the 2019 Ghana Malaria Indicator Survey dataset. The data analysed were restricted to women aged 15–49 years who reported having a live birth within the two years preceding the survey. A modified Poisson regression model was used to determine factors associated with SP uptake during pregnancy. Geospatial analysis was employed to map the spatial distribution of optimal SP uptake across the ten regions of Ghana using R software.

**Results:**

The likelihood that pregnant women received optimal SP correlated with early initiation of first antenatal care (ANC), number of ANC contacts, woman’s age, region of residence, and family size. Overall, the greater the number of ANC contacts, the more likely for pregnant women to receive optimal SP. Women with four or more ANC contacts were 2 times (aPR: 2.16; 95% CI: [1.34–3.25]) more likely to receive optimal SP than pregnant women with fewer than four ANC contacts. In addition, early initiation and a high number of ANC contacts were associated with a high number of times a pregnant woman received SP. Regarding spatial distribution, a high uptake of optimal SP was significantly observed in the Upper East and Upper West Regions, whereas the lowest was observed in the Eastern Region of Ghana.

**Conclusions:**

In Ghana, there were regional disparities in the uptake of SP during pregnancy, with the uptake mainly correlated with the provision of ANC services. To achieve the country’s target for malaria control during pregnancy, there is a need to strengthen intermittent preventive treatment for malaria during pregnancy by prioritizing comprehensive ANC services.

## Background

Malaria during pregnancy is a major public health challenge, especially in low- and middle-income countries (LMICs). It is the most threatening and prevalent infection in sub-Saharan Africa (SSA), where *Plasmodium* (P.) *falciparum* is the most common malaria parasite [1]. It is also dominant in some regions of Southeast Asia, the Western Pacific, and South America, where P. *vivax* and P. *falciparum* are the leading causes of the disease [1]. In 2020, out of 121.9 million pregnancies at risk of malaria globally, the World Health Organization (WHO) region with the highest number of pregnancies at risk of malaria was Southeast Asia, with 52.9 million pregnancies, followed by the African region with 46.1 million pregnancies, and the Eastern Mediterranean region with 11.1 million pregnancies [2]. In 2021, out of 40 million pregnancies in high-to-moderate transmission countries in SSA, nearly 13.3 million (32%) women experienced malaria infection during pregnancy [3]. An estimated 6.5 million malaria infections during pregnancy were reported in West Africa, which represents almost 50% of all malaria cases during pregnancy in SSA [3].

Malaria during pregnancy is associated with poor maternal, foetal, and neonatal health outcomes, including severe malaria, preterm birth (PTB), low birth weight (LBW), stillbirth, impaired child development, and risk for maternal and neonatal mortality [4–6]. In a systematic review of African and Western Pacific studies, Cates et al. [7] found higher odds of delivering babies with LBW among women with malaria at antenatal care (ANC) enrolment and during childbirth than among uninfected women. Furthermore, another systematic review to determine the contribution of malaria during pregnancy to birth outcomes revealed overall LBW and PTB risks of 63% and 23%, respectively [8]. In addition to the effects of malaria during pregnancy on prematurity and LBW, prenatal malaria exposes the foetus to congenital malaria and thus early malaria infection as well as early impaired development [9]. These adverse outcomes of malaria during pregnancy occur mostly in SSA, where the burden of malaria is high. For instance, there were 961,000 newborns with LBW in 2021 due to malaria during pregnancy, and the West Africa subregion accounted for at least half (48.5%) of these cases [3].

To prevent malaria infection during pregnancy and its consequences, the WHO recommended intermittent preventive therapy with sulfadoxine-pyrimethamine (IPTp-SP) in addition to vector control strategies [10]. IPTp-SP has been shown to be the most cost-effective intervention for preventing malaria during pregnancy and its consequences. According to a systematic review conducted in 2020, IPTp-SP was the most cost-effective intervention for preventing LBW, maternal anaemia, and disability-adjusted life-years related to malaria during pregnancy [11]. Furthermore, chemoprophylaxis with sulfadoxine-pyrimethamine (SP) during pregnancy has been shown to reduce the risk of antenatal parasitaemia during the first and second pregnancies as well as premature birth and placental parasitaemia [12]. With these advantages, every pregnant woman is expected to benefit from SP; however, evidence show that only 35% of pregnant women in SSA received three doses of SP in 2021 [3].

In Ghana, the prevalence of asymptomatic *P. falciparum* infection among pregnant women was between 13 and 26% in the northern savannah zone, between 5 and 20% in the middle transitional/forest zone, and between 5 and 11% in the coastal savannah area in 2019 [13]. Furthermore, a study using national data from the District Health Information Management System II (DHIMS II) indicated that the prevalence of malaria during pregnancy was 34.3% for all P. species in 2021 [14]. Evidence from some studies conducted in Ghana suggests that the uptake of SP during pregnancy is linked to positive maternal and newborn outcomes, such as improved birth weight, fewer newborn complications, and an increased likelihood of term birth [15, 16]. However, the Ghana Demographic and Health Survey 2022 (GDHS 2022) reported that only 60% of pregnant women had received optimal SP (three or more doses) during their most recent pregnancy [17]. Despite this progress, providing long-lasting insecticide treated nets to first-time mothers and a minimum of three doses of SP to all pregnant women attending ANC services will continue improving maternal and birth outcomes in Ghana [18, 19].Previous studies conducted in Ghana have shown a correlation between SP uptake during pregnancy and several factors [20–24]. For instance, De-Gaulle et al. (2022) found that untrained health staff and stock-outs decreased SP uptake, while in a study by Kumah et al. (2022), the number of ANC contacts, women’s educational level, and time to the first ANC contact were found to be predictive factors of SP uptake. However, these studies were restricted to the district or regional level and suggested a large sample size to generalize the findings to all of Ghana. Malaria-transmission is highly heterogeneous both spatially and temporally, and this distribution has been used to identify places requiring targeted interventions to reduce the malaria burden [25, 26]. Although Malaria is endemic in Ghana, there are regional variations among the three ecological zones (Northern, Middle, and Coastal), with the northern zone showing a disproportionately high prevalence of disease during pregnancy compared to other zones [13]. Therefore, a comprehensive understanding of SP uptake and its spatial distribution will contribute to tailored interventions with the aim of increasing the coverage and uptake of SP during pregnancy. This study sought to map the geospatial distribution of optimal SP uptake and identify factors associated with SP uptake during pregnancy in Ghana.

## Methods

### Study design, setting, and data source

The primary survey was conducted in Ghana, a lower- middle-income country in West Africa. Ghana is a perennial malaria endemic country with a population of approximately 32 million and an overall malaria prevalence of 8.6% in 2023 [19]. As of December 2018, Ghana had 10 administrative regions, and the survey was conducted in all 10 administrative regions because the boundaries of the current 16 regions were not available during the survey. This study was based on nationally representative cross-sectional datasets from the 2019 Ghana Malaria Indicator Survey (GMIS-2019). The GMIS-2019 collected data related to background characteristics, reproductive history, and malaria indicators among women aged 15–49 years [27]. The data were collected from September to November 2019. After receiving an explicit authorization to use the GMIS-2019 dataset, the dataset was downloaded from the Demographic and Health Survey (DHS) program website (www.dhsprogram.com).

### Sampling procedures and data collection

The GMIS-2019 followed and used the 2010 Ghana Population and Housing Census to identify all census enumeration areas (EAs). An EA is the smallest geographical area to be canvassed during a survey, and its location, kind of dwelling, and estimated population are known. Although 16 regions have existed since 2018, the sampling was based on the 10 administrative regions for which boundaries were available during the survey [28]. The 10 regions were divided into rural and urban EAs, yielding 20 strata. The GMIS-2019 used two-stage sampling to identify participating households. In the first stage, two hundred (200) EAs were randomly selected with a probability proportionate to their size (of which 103 and 97 were rural and urban, respectively); additionally, a listing of households was conducted to identify potential households in the second stage. For the second stage, a systematic selection of 30 households per EA was conducted, totalling 6,000 households, 3,090 of which were from rural areas and 2,910 of which were from urban settings. However, a total of 5,833 households were occupied during the survey, and only 5,799 households were successfully interviewed (response rate of 99%). Prior to data collection, fieldworkers were trained for 3 weeks, and the data were collected over a period of 8 weeks from 25 September to 24 November 2019. All women aged between 15 and 49 years, permanent residents or visitors who stayed in the selected household the night before the survey were eligible for the interview. A structured questionnaire including questions related to household and woman-related data was used. The recorded data included sociodemographic characteristics, reproductive and pregnancy history, malaria indicator information, and household characteristics to determine wealth status as well as ownership and utilization of mosquito bed nets. Furthermore, the survey collected fieldworker background and biological samples for anaemia and malaria tests from participating children aged 6–59 months within each household.

### Study sampling and population

A total of 5,246 women aged 19–49 years were scheduled for interviews, but only 5,181 participated in the GMIS-2019. The study sample of this analysis consisted of all women aged 15–49 years who participated in the GMIS-2019 and reported a livebirth within the 2 years preceding the survey. The sample was based on the definition of intermittent preventive treatment coverage, which concerns women who gave a live birth in the previous two years [29]. A total of 1,262 women reported having a live birth within the 2 years preceding the GMIS-2019. In addition to not de jure residents (not permanent residents), women who reported that they did not know the number or if they took SP during pregnancy were excluded. Therefore, the total sample consisted of 1,224 women aged 15 to 49 years.

### Study variables

#### Dependent variable

The outcome variables were the number of times a pregnant woman took SP and the optimal dose (3 or more) of SP taken during the last pregnancy (details in Table 1). The dependent variables were created using the following GMIS-2019 questions related to the last pregnancy that resulted in a live birth: “*During this pregnancy, did you take SP/Fansidar to keep you from getting malaria?”*, and “*How many times did you take SP/Fansidar during this pregnancy?”.*

### Independent variables

The selection and categorization of independent variables were based on existing evidence and their availability in the dataset (details in Table 1).

**Table 1:** Variables

Variables	Measurement	Operational Definitions
**Optimal SP uptake**	**Categorical**	**Received 3 or more doses of SP during pregnancy**
**Times taken SP**	**Numerical**	**Number of times a pregnant woman took SP**
Source of SP	Categorical	The way used to get SP
Age (years)	Categorical	Age of women during the survey
Region	Categorical	The region where the women reside
Residence	Categorical	The residence area of a woman
Education level	Categorical	Women’s highest level of education
Religion	Categorical	Women’s religion
Ethnicity	Categorical	This is the ethnicity of women
Household size	Categorical	Total number of members in a household
Sex of household head	Categorical	The sex of the household head
Literacy	Categorical	The reading ability of the women
Household wealth index	Categorical	The economic status of the women based on household assets and possessions
Parity	Categorical	Number of children ever born to a woman
ANC initiation time	Categorical	The time a pregnant woman commenced the first ANC during pregnancy
Frequency of ANC contacts	Categorical	The number of ANC attended by a pregnant woman
ITNs possession	Categorical	The household has a mosquito bed net for sleeping
ITNs use	Categorical	Women slept under a bed mosquito net the night before the interview
Health insurance coverage	Categorical	Women who are covered by any form of health insurance
Awareness of malaria care covered by health insurance	Categorical	The awareness that malaria care is covered under health insurance
Exposure to malaria messages	Categorical	The women heard or seen any malaria messages in the past 6 months

*Note* The dependent variable is in bold, SP: Sulfadoxine-pyrimethamine; ANC: Antenatal care; ITN: Insecticide-treated nets

### Data management and analysis

The data were extracted, cleaned, recoded, and analysed using Stata version 17 statistical software. The analysed data were restricted to women aged 15–49 years who reported having a live birth within the 2 years preceding the GMIS-2019. Furthermore, women who were not permanent residents and who did not know the number of doses taken or if they took SP/Fansidar were excluded. For statistical analysis, the uptake of optimal SP during pregnancy was categorized and coded into 0 = less than 3 doses (< 3) and 1 = at least 3 doses (≥ 3). Some independent variables were recoded for this study analysis. The original dataset contained 10 categories of religion. To avoid enlargement, the women were categorized as Christian, Islamic, other, or not religious. A woman was Christian if she was Catholic, Presbyterian, Methodist, Anglican, Pentecostal/Charismatic or other Christian, while spiritualists and traditional were categorized into other religions. The source of SP was other services if the woman received the SP outside ANC contacts (other health facility visits or from another source). For the literacy variable, a woman was considered literate if she was able to read the given whole sentence and illiterate otherwise. Age was first categorized into 15–19, 20–24, 25–29, 30–34, 35–39, 40–44, and 45–49 years in line with standard reporting for women in their reproductive age. However, it was recategorized into 15–24 years, 25–34 years, and 35–49 years due to few observations in some of the age categories during analysis.

The ANC number and the first ANC start time were categorized based on WHO-focused ANC, which recommends a minimum of 4 ANC contacts, with the first ANC occurring between 8 and 12 weeks of pregnancy [30]. Similarly, parity was categorized according to the fertility rate of 4 children per woman according to the 2014 Ghana DHS [31]. Therefore, family size was also categorized into less than or equal to six members (4 children + two parents) and greater than 6. The survey design features (stratification, clustering) and sampling weighting were performed. Three levels of weighting were used. First, individual-level weighting was performed, which was the inverse of the product of the probabilities of selection of an EA, selection of a household within a selected EA and the probability of selection of a woman aged 15–49 in a household. The second level weighting was the population distribution, which is the inverse of the population of the age categories within the survey and the population representation in Ghana. The final weighting was the non-response rate, which was computed as the inverse of the number selected for each age category over the actual participants. The final weight was determined as the product of the individual weights, population distribution weights and the non-response rate. During analysis, the “svy” command was used to address the complex study design.

For descriptive statistics, cross-tabulation was used to summarize the frequencies and percentages of participants’ background characteristics. For the statistical analysis, a modified Poisson regression model was used to determine the factors associated with SP uptake (optimal SP and number of times SP was taken) [32]. Prior to the final model, a univariable Poisson regression was carried out to determine the unadjusted prevalence ratio (PR) of each independent variable for optimal SP uptake and the number of times a pregnant woman received SP. All independent variables with *p* < 20% were candidates for the multivariable Poisson regression model [33]. The final model was constructed with a manual backward selection process to identify which factors impacted SP uptake during pregnancy (the optimal SP and number of times SP was taken). The Akaike’s information criterion (AIC) was used, and the best model was the one with the lowest AIC [34]. Before spatial distribution mapping, the weighted prevalence of optimal SP uptake per region was exported from Stata to Excel and then imported into R Studio. The shapefile was subsequently merged with the optimal SP uptake for each region using R software version 4.3.0. For spatial analysis, autocorrelation analysis (global Moran’s I) was performed in R to determine whether nearby regions tend to exhibit similar prevalences of optimal SP uptake [35]. Moran’s I values vary from − 1 to + 1, and values near − 1 indicate that regions with low uptake are located near other regions with high uptake; values around + 1 suggest concentrations of regions with a similar prevalence of optimal SP uptake. A statistically significant Moran’s I (*p* < 0.05) indicates that spatial factors are associated with the optimal SP uptake.

## Results

### Sociodemographic characteristics of women aged 15–49 years who reported a live birth within 2 years before the GMIS-2019

The descriptive statistics of the sociodemographic characteristics of the participating women are presented in Table 2. The study included 1,224 women between the ages of 15 and 49 years who reported that they had given birth to a live child in the two years preceding the GMIS-2019. Most of the participating women were Christians and from families with male household heads. Additionally, 80% had no more than 4 children, and 67.5% were from families with fewer than 7 members. Of these women, nearly 56% had no formal education and were rural residents, 53% had a secondary education level, and 50% were aged 25–34 years. The wealth status of a woman’s family was almost evenly distributed across wealth index quintile categories ranging from 23% for women coming from poor families to 16.6% in the richest families.

Approximately 90% of women attended at least 4 ANC services. Additionally, 64% of women initiated their first ANC during the first trimester of pregnancy, and less than 2% of those women did so during the third trimester. Although most households owned mosquito bed nets for sleeping, only 61% of women slept under mosquito bed nets the night before GMIS-2019. Furthermore, 92% of women were covered by any health insurance, whereas only three-quarters of women reported being aware that health insurance covers care for malaria. Additionally, approximately two-thirds of women reported having seen or heard any malaria messages in the six months prior to the survey. More than 99% of women received their SP during ANC services, while less than 1% received their SP from other services.

**Table 2:** Sociodemographic characteristics of women aged 15–49 years with a live birth within 2 years, GMIS 2019 (*N* = 1,224)

Explanatory variables	Frequency*	Percentage (%)*
Age group (x̄: 28.6, SE: 0.2)		
15–24 years	382	28.9
25–34 years	593	50.2
35–49 years	249	20.9
Education level		
None	281	19.3
Primary	275	21.9
Secondary	604	53.2
Tertiary	64	5.6
Religion		
Christianity	889	76.1
Islam	288	20.5
Other	16	0.9
No religion	31	2.5
Ethnicity		
Akan	412	40.5
Ga/Dangme	58	5.9
Ewe	155	14.9
Mole Dagbani	395	22.3
Other	204	16.4
Region		
Western	116	9.9
Central	122	8.3
Greater Accra	101	14.6
Volta	109	10.8
Eastern	89	9.4
Ashanti	123	16.6
Brong Ahafo	111	8.4
Northern	187	13.9
Upper East	130	4.7
Upper West	136	3.4
Residence		
Urban	482	43.4
Rural	742	56.6
Family size (x̄: 6.0, SE: 0.1)		
1–6 members	785	67.5
>6 members	439	32.5
Household head sex		
Male	893	70.3
Female	331	29.7
Literacy		
Illiterate	730	56.6
Literate	494	43.4
Wealth index quintile		
Poorest	371	20.7
Poor	280	23.1
Middle	235	19.8
Richer	188	19.8
Richest	150	16.6
Parity (x̄: 3.0, SE: 0.1)		
0–4 Children	982	80.1
> 4 Children	242	19.9
Time of first ANC **		
1st trimester	790	65.5
2nd trimester	382	32.7
3rd trimester	25	1.8
Number of ANC (x̄: 6.9, SE: 0.1)		
0–3 ANC	118	10.2
≥4 ANC	1106	89.8
Possession of ITN		
No	142	12.4
Yes	1082	87.6
ITN use		
No	426	39.0
Yes	798	61.0
Health insurance coverage		
No	90	8.0
Yes	1134	92.0
Exposure to malaria messages		
No	517	37.4
Yes	707	62.6
Aware malaria is covered by health insurance		
No	307	27.0
Yes	917	73.0
Source of SP		
ANC	1127	99.7
Other	5	0.3

*Note* *: Frequencies for a weighted sample; **: Women who attend ANC contacts are less than the sample size; ANC: Antenatal care; ITN: Insecticide-treated nets; SP: Sulfadoxine-pyrimethamine

### Factors associated with the uptake of optimal SP during pregnancy

Table 3 summarizes the results from the modified Poisson regression model, which sought to determine factors associated with optimal SP uptake among pregnant women. Overall, five variables, including woman’s age, region of residence, family size, first ANC initiation time, and number of ANC contacts during pregnancy, predicted the uptake of optimal SP. Specifically, this study revealed that, compared to women from a household size less than or equal to 6, women from a family size of more than 6 members were 11% more likely (aPR: 1.11; 95% CI: [1.00–1.25]) to receive optimal SP. Also, women aged 25–34 and 35–49 years were 16% (aPR: 1.16; 95% CI: [1.01–1.33]) and 19% (aPR: 1.19; 95% CI: [1.01–1.40]) more likely to receive optimal SP than women aged 15–24 years, respectively. According to region of residence, women from the Eastern Region were 32% (aPR: 0.68; 95% CI: [0.52–0.89]) less likely to uptake optimal SP than women from the Western Region were. Furthermore, compared to women who attended less than 4 ANC contacts, the prevalence of optimal SP uptake was 2 times greater (aPR: 2.16; 95% CI: [1.34–3.25]) among women who attended at least 4 ANC contacts during pregnancy. The likelihood of receiving the optimal SP was lower (aPR: 0.81; 95% CI: [0.71–0.92]) among women who started their first ANC during the second trimester of pregnancy than among women who initiated their first ANC during the first semester.

**Table 3:** Unadjusted and adjusted prevalence ratio of predictive factors associated with the uptake of “Optimal SP”, GMIS 2019

Explanatory variables	uPRs	*P* value	95% CI	APRs	*P* value	95% CI
Age group		0.005				
15–24 years (ref)						
25–34 years	1.19	0.014	[1.03–1.38]	1.16	0.035	[1.01–1.33]
35–49 years	1.24	0.080	[1.05–1.46]	1.19	0.031	[1.01–1.40]
Education level		0.514				
None (ref)						
Primary	0.82	0.042	[0.68–0.99]			
Secondary	0.98	0.845	[0.82–1.17]			
Tertiary	1.08	0.474	[0.86–1.35]			
Religion		0.857				
Christian (ref)						
Islam	1.11	0.087	[0.98–1.26]			
Other	1.05	0.823	[0.68–1.61]			
No religion	0.93	0.755	[0.62–1.40]			
Ethnicity		0.407				
Akan (ref)						
Ga/Dangme	0.78	0.073	[0.60–1.02]			
Ewe	0.92	0.388	[0.77–1.10]			
Mole Dagbani	1.02	0.681	[0.90–1.16]			
Other	0.90	0.334	[0.72–1.11]			
Region		0.085				
Western (ref)						
Central	0.95	0.589	[0.79–1.14]	0.92	0.414	[0.76–1.11]
Greater Accra	0.90	0.349	[0.73–1.11]	0.86	0.210	[0.69–1.08]
Volta	0.80	0.250	[0.55–1.16]	0.77	0.103	[0.57–1.05]
Eastern	0.66	0.003	[0.50–0.87]	0.68	0.006	[0.52–0.89]
Ashanti	0.97	0.754	[0.81–1.15]	0.95	0.628	[0.79–1.14]
Brong Ahafo	0.96	0.767	[0.77–1.20]	0.97	0.789	[0.79–1.18]
Northern	0.97	0.842	[0.77–1.23]	0.94	0.500	[0.78–1.12]
Upper East	1.16	0.083	[0.97–1.39]	1.06	0.518	[0.88–1.28]
Upper West	1.20	0.034	[1.01–1.42]	1.19	0.052	[0.99–1.43]
Residence		0.884				
Urban (ref)						
Rural	1.00	0.884	[0.89–1.14]			
Family size		0.085				
1–6 members (ref)						
>6 members	1.10	0.085	[0.98–1.24]	1.11	0.049	[1.00–1.25]
Household head sex		0.112				
Male (ref)						
Female	0.89	0.112	[0.78–1.02]			
Literacy		0.435				
Illiterate (ref)						
Literate	1.05	0.435	[0.92–1.20]			
Wealth index quintile		0.668				
Poorest (ref)						
Poor	1.04	0.643	[0.87–1.23]			
Middle	1.04	0.631	[0.86–1.26]			
Richer	1.06	0.509	[0.88–1.29]			
Richest	1.03	0.736	[0.83–1.29]			
Parity		0.875				
0–4 Children (ref)						
> 4 Children	1.01	0.875	[0.86–1.18]			
Time of first ANC		< 0.001				
1st trimester (ref)						
2nd trimester	0.73	< 0.001	[0.63–0.84]	0.81	0.001	[0.71–0.92]
3rd trimester	0.52	0.025	[0.29–0.92]	0.90	0.736	[0.51–1.60]
Number of ANC		< 0.001				
0–3 ANC (ref)						
≥4 ANC	2.40	< 0.001	[1.60–3.60]	2.16	< 0.001	[1.44–3.25]
Possession of ITN		0.673				
No (ref)						
Yes	0.95	0.673	[0.78–1.17]			
ITN use		0.989				
No (ref)						
Yes	0.99	0.989	[0.88–1.13]			
Health insurance coverage		0.069				
No (ref)						
Yes	1.31	0.069	[0.97–1.77]	1.13	0.268	[0.90–1.41]
Exposure to malaria messages		0.014				
No (ref)						
Yes	1.13	0.014	[1.02–1.26]	1.07	0.119	[0.98–1.17]
Aware malaria is covered by health insurance		0.186				
No (ref)						
Yes	1.12	0.186	[0.94–1.32]			
Source of SP		0.642				
ANC (ref)						
Other	0.84	0.642	[0.42–1.69]			

*Note* uPR: Unadjusted prevalence ratio; aPR: Adjusted prevalence ratio; ref: reference; ANC: Antenatal care; ITN: Insecticide-treated nets; SP: Sulfadoxine-Pyrimethamine

### Factors associated with the number of times a pregnant woman received SP

The number of times a pregnant woman received SP is likely to increase with a high number of ANC contacts as well as early initiation of ANC contacts during pregnancy (Table 4). Pregnant women who attended at least four ANC contacts were 1.52 times (aPR: 1.52; 95% CI: [1.28–1.80]) more likely to receive SP than women with less than four ANC. Pregnant women who initiated their first ANC during the first term of pregnancy had 1.10 times (aPR: 0.90; 95% CI: [0.85–0.98]) higher prevalence of receiving SP compared to women who started their first ANC during the second term of pregnancy.

**Table 4:** Unadjusted and adjusted prevalence ratio of factors associated with the number of times a pregnant woman received SP, GMIS-2109

Explanatory variables	uPRs	*P* value	95% CI	APRs	*P* value	95% CI
Age group		0.091				
15–24 years (ref)						
25–34 years	1.06	0.134	[0.97–1.16]			
35–49 years	1.07	0.112	[0.98–1.18]			
Education level		0.233				
None (ref)						
Primary	0.96	0.528	[0.86–1.08]			
Secondary	1.05	0.478	[0.91–1.20]			
Tertiary	1.11	0.239	[0.92–1.34]			
Religion		0.062				
Christian (ref)						
Islam	1.02	0.476	[0.95–1.10]			
Other	0.96	0.857	[0.68–1.37]			
No religion	0.77	0.053	[0.59–1.00]			
Ethnicity		0.668				
Akan (ref)						
Ga/Dangme	0.92	0.385	[0.76–1.10]			
Ewe	0.97	0.658	[0.86–1.09]			
Mole Dagbani	0.98	0.647	[0.91–1.05]			
Other	0.95	0.566	[0.82–1.11]			
Region		0.982				
Western (ref)						
Central	0.99	0.992	[0.87–1.14]			
Greater Accra	0.94	0.448	[0.81–1.09]			
Volta	1.01	0.886	[0.79–1.30]			
Eastern	0.78	0.014	[0.65–0.95]			
Ashanti	0.94	0.343	[0.84–1.05]			
Brong Ahafo	0.99	0.948	[0.85–1.15]			
Northern	0.91	0.315	[0.76–1.08]			
Upper East	1.06	0.262	[0.95–1.19]			
Upper West	1.09	0.134	[0.97–1.22]			
Residence		0.882				
Urban (ref)						
Rural	1.00	0.882	[0.91–1.10]			
Family size		0.455				
1–6 members (ref)						
>6 members	1.02	0.455	[0.95–1.11]			
Household head sex		0.079				
Male (ref)						
Female	0.92	0.079	[0.84–1.00]	0.93	0.090	[0.86–1.01]
Literacy		0.609				
Illiterate (ref)						
Literate	1.02	0.609	[0.93–1.12]			
Wealth index quintile		0.444				
Poorest (ref)						
Poor	1.11	0.106	[0.97–1.28]			
Middle	1.08	0.179	[0.96–1.21]			
Richer	1.09	0.146	[0.96–1.24]			
Richest	1.08	0.285	[0.93–1.24]			
Parity		0.596				
0–4 Children (ref)						
> 4 Children	1.02	0.596	[0.93–1.12]			
Time of first ANC		< 0.001				
1st trimester (ref)						
2nd trimester	0.87	< 0.001	[0.81–0.93]	0.91	0.016	[0.85–0.98]
3rd trimester	0.65	0.012	[0.46–0.90]	0.85	0.355	[0.61–1.19]
Number of ANC		< 0.001				
0–3 ANC (ref)						
≥4 ANC	1.63	< 0.001	[1.28–2.08]	1.52	< 0.001	[1.28–1.80]
Possession of ITN		0.947				
No (ref)						
Yes	0.99	0.947	[0.88–1.11]			
ITN use		0.524				
No (ref)						
Yes	1.02	0.524	[0.94–1.12]			
Health insurance coverage		0.109				
No (ref)						
Yes	1.17	0.109	[0.96–1.43]	1.04	0.153	[0.98–1.10]
Exposure to malaria messages		0.010				
No (ref)						
Yes	1.08	0.010	[1.02–1.15]			
Aware malaria is covered by health insurance		0.522				
No (ref)						
Yes	1.04	0.522	[0.91–1.19]			
Source of SP		0.845				
ANC (ref)						
Other	0.98	0.845	[0.83–1.16]			

*Note* uPR: Unadjusted prevalence ratio; aPR: Adjusted prevalence ratio; ref: reference; ANC: Antenatal care; ITN: Insecticide-treated nets; SP: Sulfadoxine-Pyrimethamine

### Geospatial distribution of optimal sulfadoxine-pyrimethamine uptake

**Fig. 1:**
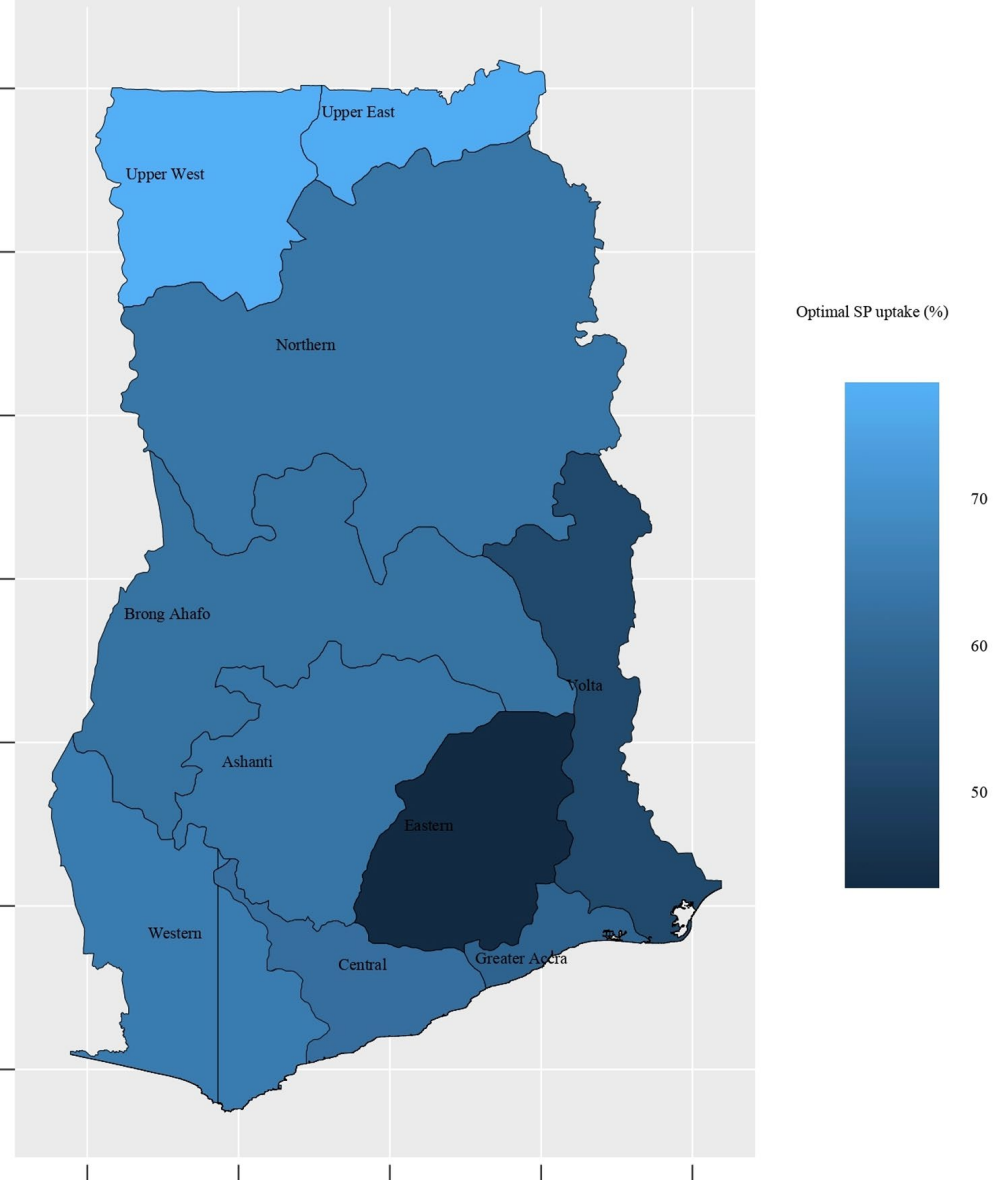
Distribution of optimal SP uptake per region

According to Fig. 1, the proportion of women who received the optimal SP varies across regions. Generally, 61% of women received the optimal SP during pregnancy. However, there are differences in terms of uptake when considering each region independently. High optimal SP uptake is statistically observed in the Upper West and Upper East Regions, where more than 70% of women received at least three doses. The lowest SP uptake was observed in the Eastern Region, at less than 50%. In the remaining Regions, nearly 60% of women had received the optimal SP, except in the Volta Region, where approximately 55% of women had received the optimal SP. Spatial autocorrelation analysis revealed the presence of spatial factors that are associated with the distribution of optimal SP uptake across regions (Moran I = 0.40, *p* < 0.01). Given that *p* < 0.01, less than 1% of the distribution of optimal SP uptake per region was due to chance.

## Discussion

Overall, the findings of this study showed that family size, woman’s age, region of residence, number of ANC contacts, and first ANC initiation time affected the likelihood of pregnant women receiving optimal SP. These results are consistent with existing evidence, especially from LMICs. The findings revealed that women with four or more ANC contacts during pregnancy had higher odds of receiving optimal SP than women who attended fewer than four ANC contacts. This positive correlation between ANC numbers and optimal SP uptake aligns with the literature emphasizing the importance of ANC services in promoting SP use. For instance, pregnant women who attended four or more ANC contacts had a high likelihood of receiving the optimal SP in studies conducted in Uganda [36], Kenya [37], and Nigeria [38]. Similar findings have been reported in previous studies, which revealed that more ANC contacts increase the odds of SP uptake [20, 39]. Similarly to the optimal SP uptake, the number of ANC contacts predicted the number of times a pregnant woman received SP. Women with four or more ANC contacts during pregnancy were more likely to receive SP many times than women who attended fewer than four ANC contacts. These results are similar to those of a study conducted in Nigeria, which found that women who attended more ANC contacts had received SP many times [38]. Similar findings have been reported in other previous studies, which revealed that more ANC contacts were more common for pregnant women who received SP many times [37, 39]. Pregnant women who attend ANC contacts have ample opportunities to interact with healthcare providers, which increases the likelihood that they may receive SP as a component of ANC services. There is evidence to support the premise that more ANC contacts is associated with high uptake of SP [30]. Although the WHO recommends administering SP from the second term of pregnancy, initiation of the first ANC during the first term of pregnancy was positively associated with optimal SP uptake. Furthermore, the number of times a pregnant woman received SP was predicted by the timing of the first ANC initiation. Women who initiated their first ANC during the first term of pregnancy were more likely to receive SP than women who started their first ANC during the second term of pregnancy were. The number of ANC contacts is also related to the early initiation (within the first trimester) of ANC. Since it is the main channel used to deliver SP, it is important to promote the early initiation of ANC during pregnancy. A study carried out in Kenya showed that women who initiated their first ANC contact after 16 weeks of gestation had a lower likelihood of receiving optimal SP than women who initiated their first ANC contact before 16 weeks of pregnancy [37]. Comparable results were also found in Tanzania [40]. However, these findings are not consistent with those of a study conducted among 4,772 Nigerian women of reproductive age, in which those who started their first ANC during the second trimester had greater odds of receiving optimal SP than did those who started their first ANC during the first trimester [41]. These findings should be understood since ANC services are an important platform for delivering SP, and early ANC initiation allows health professionals to promote and provide information about pregnancy early, including the use and benefits of IPTp-SP.

The findings also showed that women from a household with more than six members were more likely to receive optimal SP. In contrast, a study in Guinea established that women from a family of 2 to 5 members were more likely to complete malaria preventive measures, including ITN use and SP uptake, compared to women from a household with more than five members [42]. A negative correlation between family size and the use of malaria preventive measures was also established in a study conducted in Tanzania [43]. With respect to age, this study revealed a positive association between age and optimal SP uptake. The increase in optimal SP uptake was associated with increasing age. Similarly, a study in Tanzania has established that being aged 20–34 years and older than 34 years was associated with a high likelihood of receiving SP during pregnancy [40]. However, the odds of optimal SP uptake decreased with increasing age in a study conducted in Uganda [44]. In this study, Ugandan women aged more than 34 years were less likely to receive optimal SP. The conflicting results in terms of women’s age and household size could be explained by differences in pregnancy experience among women within different settings. It is expected that older women, especially those who had previous pregnancies, are more exposed to knowledge and benefits about IPTp-SP and therefore have better healthcare-seeking behaviours. Furthermore, regional residence predicted the optimal SP uptake among pregnant women. The odds of optimal SP uptake were lower for women from the Eastern Region than for women from the Western Region. These findings are in line with Solanke et al. (2023)’s findings in Nigeria, where pregnant women from southern geo-political zones were more likely to receive optimal SP. The same results were obtained in a study conducted in Tanzania [45]. The regional disparities could be explained by different initiatives to reduce malaria incidence in regions, especially those with a high prevalence of malaria. The findings of the present study demonstrated that optimal SP uptake varies significantly by region, with the highest uptake occurring in the northern part of Ghana. According to these findings, women from the Upper West and Upper East Regions had high optimal SP uptake, with more than 70% receiving the optimal SP. These regional disparities are consistent with findings from a previous study by Owusu-Boateng et al. (2017), which showed that community-based education and maternal interventions improved the utilization of maternal healthcare services, including IPTp, in northern Ghana. These regional disparities are also consistent with the results reported in two recent studies in Nigeria [38] and Guinea [42]. For the study in Nigeria, the highest optimal SP uptake was observed in the South‒East and South‒South Regions, while the lowest was observed in the North‒East and North‒West Regions. These regional disparities can be attributed to differences in the programs and activities used to reduce the prevalence of malaria in the northern part of Ghana. The lowest prevalence observed in the Eastern Region, with less than 50% of the optimal SP uptake, is a concerning finding. Additionally, Volta, an Eastern bordering Region, has a lower prevalence of optimal SP uptake (approximately 55%), which is slightly lower than the national prevalence. These findings are consistent with the findings of a study exploring regional variations in optimal SP uptake in Zambia, which revealed a lower prevalence of SP uptake in the western and southern provinces [46]. Spatial autocorrelation analysis revealed a significant correlation between spatial factors and optimal SP uptake across different Regions. In addition to spatial factors, the lower optimal SP uptake in the Volta and Eastern Regions could be explained by disparities in maternal healthcare service accessibility and utilization, including IPTp-SP.

Briefly, these findings did not challenge the existing evidence on the determinants of receiving SP during pregnancy. However, this study did not find an association between SP uptake and several factors, such as residence area, sex of household, household wealth index, possession of ITNs, use of ITNs, health insurance coverage, and exposure to malaria messages. Some of these factors, even if they were not significant in this study, have been cited by many authors [41, 42, 47–49]. For instance, contrary to the findings of Figueroa-Romero et al. (2022); Gutman et al. (2021); Okedo-Alex et al. (2020); Okeke Kalu et al. (2022), the source of SP did not predict the uptake of SP. Nevertheless, there is not enough evidence to support the effect of the source of SP since most women in this study received SP from ANC services.

### Strengths and limitations

This study’s strength is that it used a nationally representative sample obtained using multistage sampling procedures. Therefore, the findings can be applied to the entire population of Ghana. Additionally, the DHS dataset used was based on a standardized questionnaire, and only data from the most recent pregnancy within two years were included, which could minimize recall bias. As limitations, the analysis was restricted to the variables available in the GMIS-2019 questionnaire, which did not include other factors, such as health system factors, that can affect IPTp-SP. Additionally, the survey was self-reported, and women’s responses might not accurately reflect the issues as they are.

## Conclusions

This study highlighted key factors that can inform policy makers in planning and implementing programs to increase the uptake of SP in Ghana. Since taking SP was mainly associated with ANC services, the main platform for delivering SP during pregnancy in Ghana, it is recommended that the Ghana Health Service adopt a strategy to promote holistic ANC services and intensify health education on the early initiation of ANC during pregnancy to increase the uptake of SP. In addition, spatial autocorrelation analysis indicated that regional disparities are not random but are associated with spatial factors. Future research should explore the spatial factors that contribute to region-specific low uptake of SP during pregnancy, especially in the Eastern Region.

## Data Availability

The datasets analyzed during the current study are available from the DHS program website and the corresponding author (including commands in STATA and R).

## Abbreviations

ANC: Antenatal Care
DHS: Demographic and Health Survey
EAs: Census Enumeration Areas; GMIS-2019
GMIS-2019: Ghana Malaria Indicators Survey 2019
IPTp-SP: Intermittent Preventive Treatment with sulfadoxine-pyrimethamine
GMIS-2019: Ghana Malaria Indicators Survey 2019
LBW: Low-Birth Weight
LMICs: Low- and middle-income countries
GMIS-2019: Ghana Malaria Indicators Survey 2019
P: Plasmodium
PR: Prevalence ratio
PTB: Preterm Birth
SP: sulfadoxine-pyrimethamine
WHO: World Health Organization.
